# Androgen-armoured amazons: reversed sex roles in coucals are associated with testosterone in females but not males

**DOI:** 10.1098/rspb.2022.2401

**Published:** 2023-03-29

**Authors:** Wolfgang Goymann

**Affiliations:** ^1^ Max Planck Institute for Biological Intelligence, Eberhard-Gwinner-Straße 6a, D-82319 Seewiesen, Germany; ^2^ Coucal Project, PO Box 26, Chimala, Mbeya, Tanzania

**Keywords:** testosterone, progesterone, corticosterone, mating system, sex roles, polyandry

## Abstract

In some species, sexual selection is stronger in females than males. In classically polyandrous birds, for instance, females compete for mating opportunities and males care for offspring. Sex steroids such as testosterone have been suggested to regulate the behaviours of ‘role-reversed’ females and males, but comparative studies did not find evidence for a role of testosterone in relation to sex roles. However, the large variability of hormone measurements across laboratories may prevent detecting subtle differences in hormone levels. To circumvent this caveat, I compared sex steroid concentrations of females and males of two closely related and cohabiting species with different mating systems: the classically polyandrous black coucal (*Centropus grillii*) and the monogamous white-browed coucal (*C. superciliosus*). Baseline and gonadotropin-releasing hormone (GnRH)-induced testosterone concentrations were twice as high in female black coucals than female white-browed coucals, and the low pre-breeding progesterone concentrations of female black coucals were consistent with progesterone's modulatory role during agonistic interactions in this species. Baseline and GnRH-induced testosterone and progesterone concentrations did not differ between males of both species. This study provides first evidence that elevated testosterone is associated with sex-role-reversed traits in females, whereas low levels of testosterone may not be necessary to facilitate sex-role reversal in males.

## 1. Introduction

Sexual selection, or the differential access to mates or opposite-sex gametes [1], is often stronger in males than females. This bias typically results in Darwinian sex roles, which are characterized by higher competition among males for access to females, choosy females, higher variance in mating success among males, and often also a higher proportion of female than male parental care [2–9]. However, sex roles can respond flexibly to environmental and evolutionary factors, sometimes rendering sexual selection stronger in females than males [10–15]. When females compete more strongly for mates, this is commonly referred to as a ‘reversal in sex roles’ [5,15–17] (but see [18] for a criticism of the concept). In birds, higher female competition is often combined with higher male care for offspring and polyandry. A female then simultaneously or sequentially forms a ‘harem’ with several males, each of which receives a separate clutch to care for [19]. This breeding system is referred to as ‘classical polyandry’ and mainly occurs in precocial birds [19–21]. Classical polyandrous females often develop enhanced secondary sexual characteristics including weaponry, ornamentation, and they aggressively compete for resources such as territories or mates [5,22,23].

Sex steroids, such as testosterone, are key hormonal players for the development and expression of secondary sexual traits including morphology and behaviour of males (e.g. [24–29]) and females (e.g. [30–34]). In species with Darwinian sex roles, in which males are the more competitive and females are the choosier sex, testosterone levels are typically higher in males than in females. In this context, species with reversed sex roles are of special interest because they facilitate the study of competitive traits in females. Early work in birds suspected that sex-role reversal is accompanied by a reversal in sex steroid concentrations [35–37], but this was not confirmed: the hormone levels of all sex-role-reversed birds studied in their natural environment resemble those of species with Darwinian sex roles: males express higher levels of testosterone or other androgens than females [38–44]. Only a study in captive barred buttonquail (*Turnix suscitator*) failed to find differences in testosterone concentrations between females and males, with both sexes having equally low levels of this steroid [45].

Despite the overall lack of a sex reversal in sex steroid concentrations, various lines of evidence suggest that testosterone, or androgens in general, play a role in supporting ornaments or armaments of females. For instance, testosterone concentrations correlate with wing spur length in female northern jacanas [43], or throat patch size and darkness in female barred buttonquails [45]. Similarly, compared to males, the skin of female Wilson's phalaropes (*Phalaropus tricolor*) expresses higher levels of 5α-reductase, an enzyme that converts testosterone to dihydrotestosterone (DHT), another potent androgen [46]. Also, in females of species with conventional sex roles skin ornamentation is influenced by testosterone [47–49]. Female black coucals (*Centropus grillii*) and female barred buttonquail express more androgen receptors than males in parts of the social brain network [50,51]. Hence, because testosterone exerts its action by binding to such androgen receptors, the parts of the brain that are relevant for competitive and social behaviour appear to be more sensitive to testosterone in females of sex-role-reversed species. Therefore, androgenic pathways likely play a role in female armament and ornament.

Even if absolute concentrations of sex steroids are not reversed between females and males of sex-role-reversed species, females of such species may still express higher levels of testosterone than females of species with other mating systems. So far, comparative analyses of published testosterone data did not find any evidence for such a pattern [31,33,52–54], but there is also a paucity of hormone studies in females [55,56]. Similarly, males of classically polyandrous species might express lower levels of testosterone than males of species with other mating systems, since they are the only parental care providers and high levels of testosterone have been shown to suppress paternal care (summarized by [57,58]). Males of polygynous species appear to have higher testosterone peaks than males of socially monogamous species [59], but classically polyandrous males do not seem to differ from socially monogamous species [59–61]. A better predictor for male testosterone levels in birds is the rate of extra-pair paternity. Males of species with higher extra-pair paternity show higher peak levels of testosterone than males of species with little extra-pair paternity ([59]; see also [62]), suggesting a role for testosterone in sperm or mate competition.

Unfortunately, comparative data across many species are notoriously difficult to interpret because hormone levels measured in different laboratories differ in precision and accuracy. A recent study compared standardized hormone samples measured across 19 different expert laboratories and found that 80% of the variance in absolute hormone concentrations was due to laboratory identity [63]. Such a huge variability renders comparative hormone studies across laboratories especially problematic when the expected differences or effect sizes are small [64], such as when comparing the (usually low) testosterone levels of females across mating systems.

An alternative approach to large-scale across-laboratory comparisons is a more focused approach by comparing two or few closely related species that differ in a targeted trait and for which hormone concentrations have been measured in the same laboratory. Such targeted comparisons have been criticized because they statistically confound two independent variables: species membership and the biological or ecological factor in question [65]. However, because larger scale comparative studies have similar limitations we recently argued that both kinds of comparisons are necessary to advance knowledge [64]. Two- or few-species comparisons can help to formulate hypotheses and provide a first line of evidence, which can then be tested more extensively in follow-up comparisons across a larger number of species.

Therefore, I here compare sex steroid concentrations of two closely related coucal species that live in the same habitat but differ in mating systems. Coucals are Afro-Asian non-parasitic cuckoos [66,67], most of which live in socially monogamous pairs and provide biparental care. Female coucals are usually larger than males and, in general, males contribute slightly more to incubation and offspring care than females [68,69]. I investigated two coucal species that represent the taxon's two extremes with regard to sexual size dimorphism, mating system and parental care [68]. The black coucal shows the largest sexual size dimorphism in this taxon with females being 70% larger than males. Black coucals are classically polyandrous, with females singing and defending large territories, and forming polyandrous harems with up to five males, simultaneously. Males rarely sing, but each male incubates his clutch and raises the young without assistance from the female. Black coucals are partial migrants, and to the best of our knowledge, they represent the only altricial bird species with an obligatory classical polyandrous mating system [15,70–74]. The white-browed coucal (*C. superciliosus*), by contrast, is the least sexually dimorphic of all coucal species with females being only 13% larger than males and also the one with the highest similarity in sex roles. In general, white-browed coucals are socially and genetically monogamous, do not migrate, and both partners contribute similarly to territory defence and parental care [15,68, 72,73,75,76]. At my study site in the Usangu plains, Tanzania, I took advantage of the situation that both species share the same habitat, feed on the same kind of prey and often breed in close proximity to one another, thereby controlling for ecological effects on hormone levels.

If circulating testosterone concentrations play a role in classical polyandry, female black coucals should have higher circulating testosterone concentrations than female white-browed coucals. Also, since high progesterone concentrations suppress aggression in female black coucals [77], I predicted lower progesterone concentrations in female black than white-browed coucals. This should be especially the case at the beginning of the breeding season, when female black coucals establish their territories. For males, there are two alternative hypotheses. First, if high extra-pair paternity rates are connected to high levels of testosterone [59], male black coucals should have higher levels of testosterone than white-browed coucals. Extra-pair paternity is high in black coucals and basically absent in white-browed coucals [78,79], and black coucals also sire offspring in other males’ nests while tending their own young [80]. On the other hand, if testosterone suppresses paternal care [57] male black coucals should have lower levels of testosterone than white-browed coucals, because paternal care is more critical in black than white-browed coucals. This hypothesis would be also supported by the fact that black coucals, in contrast with most bird species, only develop one testis [81] and testis size has been shown to correlate with testosterone concentrations in birds [59]. To test the above hypotheses, I investigated baseline and maximum testosterone concentrations and other steroid hormones for both females and males. Maximum testosterone concentrations (i.e. the capacity to produce testosterone) were induced using injections of gonadotropin-releasing hormone (GnRH).

## 2. Methods

I studied sympatric populations of black and white-browed coucals in the grasslands of the Usangu wetland (8°41‘S 34°5‘E; 1000 m above sea level) in Mbeya Region of south-western Tanzania. Data were collected during the breeding seasons (typically January–June) from 2004 to 2022 (for details see [72]). Adult coucals were caught using mist nets (with and without conspecific playback). Because female white-browed coucals are difficult to lure to the mist net during the early breeding stages (pre-breeding and mating), sample size is lower for these breeding stages compared to other stages. Immediately after capture (mean ± s.d.: 2 : 17 ± 1 : 53 min, *n* = 479) I took a small blood sample (100–150 µl) from the wing vein for sex steroid analyses. Then birds were measured and banded with a numbered aluminium and two colour-rings for individual identification. In some years, a subset of birds (29 female and 58 male black coucals, as well as 42 female and 80 male white-browed coucals) was injected following blood sampling with 100 µl GnRH (Bachem H 3106; 7.5 µg dissolved in 100 µl isotonic saline, dosage body-size-adjusted following Jawor *et al.* [82]) into the *pectoralis major* muscle and kept in a holding bag for 30 min, after which another blood sample (50–100 µl) was taken for the measurement of GnRH-induced steroid concentrations. The timing for the second sampling was chosen based on the typical sampling schedule for such experiments in birds (i.e. [44,82–84]). Then birds were released at the site of capture.

Blood samples were processed as described in the electronic supplemental material. Radioimmunoassays of progesterone (P_4_), testosterone (T), DHT and oestradiol (E_2_; *n* = 14 assays per hormone) were performed using a modification [77] of the method established by Wingfield & Farner [85] (for details see supplementary material). The lower detection limits were 3.4 ± 1.8 pg/tube for P_4_, 0.8 ± 0.4 pg/tube for DHT, 0.5 ± 0.2 pg/tube for T and 0.3 ± 0.1 pg/tube for E_2_. Intra-assay coefficients of variation as determined by extracted chicken plasma pools were 10.3 ± 4.4% for P_4_, 1.4 ± 1.9% for DHT, 8.4 ± 7.5% for T and 9.9 ± 6.4% for E_2_. The inter-assay coefficients were 9.8% for P_4_, 9.8% for DHT, 12.0% for T and 27.7% for E_2_. All samples were above the detection limit for P_4_, DHT and T, but for E_2_ only a small fraction (6%) of all samples measured were above the detection limit. Therefore, E_2_ results were not further evaluated.

### (a) Statistical analyses

All statistical analyses were conducted with R version 4.1.2 (R Core Development Team, Vienna, 2021). To compare baseline concentrations of T, DHT and P_4_ of the two species, I used linear mixed models (R package lme4 [86]) and compared hormone concentrations separately for females and males in relation to species and breeding stage. First, I included the interaction between species and breeding stage, but for T and DHT, I dropped the interaction because the models without interaction had a better fit. The interaction was maintained for female and male P_4_ data. I distinguished four breeding stages: pre-breeding (defined as the time when birds established their territories but did not yet copulate, build nests or lay eggs), mating (defined as the period during which males typically followed females, birds frequently copulated and/or laid eggs), incubating (when birds incubated eggs) and feeding (when birds were feeding nestlings). Because female black coucals never incubate and only rarely feed nestlings [70,71,73] the sample for female black coucals basically consisted of pre-breeding and mating/egg-laying females. Since onset of the project in 2001, only three black coucal females fed nestlings (at the end of the breeding season, when there were no more males to initiate new clutches). Therefore, the dataset includes a sample of one of these females that was feeding nestlings. Before analysis, I checked if the time between capture and sampling, storing samples frozen or in ethanol, or whether the presence or absence of playback and its’ duration, or body mass explained a meaningful proportion of the variance in the data. This was not the case, however, and I therefore removed these variables from all models.

To compare baseline with GnRH-induced concentrations of hormones, I conducted mixed model analyses for each sex separately using log-transformed hormone levels as independent variables. For females, I included species, the GnRH status (before and after injection) and their interaction as fixed factors. Because most female black coucals included in the sample were either pre-breeding or mating and most female white-browed coucals were either incubating or feeding young, I did not include breeding stage, but Julian date as a covariate, instead. Individual identity was included as a random effect to control for the repeated measurement of hormone concentrations before and after GnRH injection. For males, I conducted a similar analysis but this time did not control for Julian date but instead included breeding stage (pre-breeding/mating, incubating and feeding young) as covariate. In the electronic supplementary material, I also include corticosterone concentrations measured before and after GnRH injections. Corticosterone concentrations were measured according to the method published by [87].

Model residuals were examined using graphical methods (i.e. qq plots of residuals and random effects, fitted values versus residuals) for homogeneity of variance, violation of normality assumptions or other departures from model assumptions and model fit [88]. For inferences from the models, I obtained Bayesian parameter estimates and their 95% credible intervals (using bsim of the R package arm [89] that uses a flat prior distribution [90]). Biologically meaningful differences between groups were assessed by comparing the ranges of the 95% credible intervals between groups or a regression slope. This 95% credible interval provides an estimate for the mean with a probability of 0.95. If the credible interval of one group does not overlap with the mean estimate of another group, the groups can be assumed to differ from each other. Similarly, if the 95% credible interval of the slope in a regression does not include zero, it can be assumed that there is a meaningful relationship between the continuous predictor and the dependent variable [88]. If not indicated otherwise, data are presented as individual data points in combination with posterior means and their respective 95% credible intervals (in text and tables reported within squared brackets). I also provide the posterior probability *P*(β) of the likelihood that the parameter estimates are larger than zero, with values of *P*(β) close to one indicating statistically meaningful effects or differences between groups. *P*(β) values between 0.975 and 1 are printed in italics. Finally, *R*²-values for linear models, or the respective marginal and conditional *R*²-values for mixed models were provided using the R package MuMIn [91] following [92].

## 3. Results

### (a) Baseline steroid hormone concentrations

**Figure 1.**
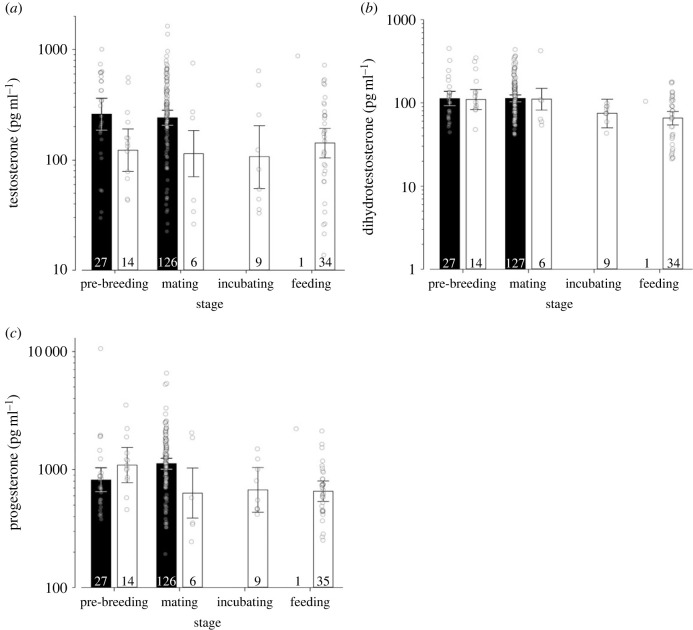
(*a*) Testosterone, (*b*) DHT and (*c*) progesterone concentrations of female black coucals (black bars) and female white-browed coucals (open bars). Bar charts with error bars represent posterior means and their 95% credible intervals, open dots refer to individual data points, and the numbers at the lower end of the bars refer to sample sizes. If the credible interval of one group does not overlap with the posterior mean of another group, the groups can be assumed to differ from each other. Please note the logarithmic scaling of the *y*-axes.

Female black coucals had consistently higher baseline levels of testosterone that were twice as high than those of female white-browed coucals (all *P*(β) > 0.99). Because female black coucals never incubate and normally also do not feed their nestlings, most data stem from pre-breeding or mating (including egg-laying) females. One female black coucal was sampled when she was feeding nestlings, a behaviour that was only seen in three females since onset of the project in 2001. The testosterone concentration of this feeding female was with 874 pg ml^−1^ higher than those of all female white-browed coucals sampled during the same breeding stage (figure 1*a*; table 1*a*; electronic supplementary material, table S1). In contrast with testosterone, DHT levels of females did not differ between species, but were slightly higher in white-browed coucals during pre-breeding and mating/egg-laying than during incubation (all *P*(β) > 0.94) and clearly higher compared to when they were feeding nestlings (all *P*(β) > 0.99; figure 1*b*; table 1*b*; electronic supplementary material, table S1). Progesterone levels of female black coucals were low during pre-breeding and higher during mating/egg-laying (*P*(β) > 0.99). By contrast, female white-browed coucals tended to have higher levels of progesterone during pre-breeding than during the other three breeding stages (all *P*(β) > 0.95); figure 1*c*; table 1*c*; electronic supplementary material, table S1).

**Table 1.** Comparison of female black and white-browed coucals (WBC). Estimates of effect sizes and the 2.5% and 97.5% quantiles (limits of the 95% Bayesian credible interval) of the posterior distribution of log hormone concentrations. *P*(β) values between 0.975 and 1 are printed in italics and indicate evidence for statistically meaningful effects.

parameter	posterior mean	2.5 % quantile	97.5 % quantile	*P*(β)
(*a*) testosterone (*R*²= 0.090, adj *R*² = 0.073; *F*_4,209_ = 5.195).				
intercept (pre-breeding)	5.558	5.225	5.890	1
*species (WBC)*	*−0.743*	*−1.224*	*−0.271*	*0.998*
stage mating	−0.073	−0.438	0.280	0.661
stage incubating	−0.139	−0.922	0.653	0.641
stage feeding	0.138	−0.393	0.666	0.703
(*b*) DHT (*R*² = 0.122, adj *R*² = 0.106; *F*_4,211_ = 7.346).				
intercept (pre-breeding)	4.722	4.524	4.923	1
species (WBC)	−0.023	−0.319	0.272	0.557
stage mating	0.213	−0.213	0.227	0.473
stage incubating	−0.386	−0.873	0.093	0.941
*stage feeding*	*−0.519*	*−0.842*	*−0.183*	*0.999*
(*c*) progesterone (*R*² = 0.133, adj *R*² = 0.108; *F*_6,209_ = 5.341).				
intercept (pre-breeding)	6.708	6.485	6.933	1
species (WBC)	0.284	−0.124	0.694	0.919
stage mating	0.311	0.056	0.563	0.992
stage incubating	−0.480	−1.041	0.061	0.957
stage feeding	0.997	−0.208	2.221	0.947
*stage × species*				
* WBC mating*	*−0.855*	*−1.498*	*−0.212*	*0.995*
* WBC feeding*	*−1.504*	*−2.805*	*−0.232*	*0.989*

**Figure 2.**
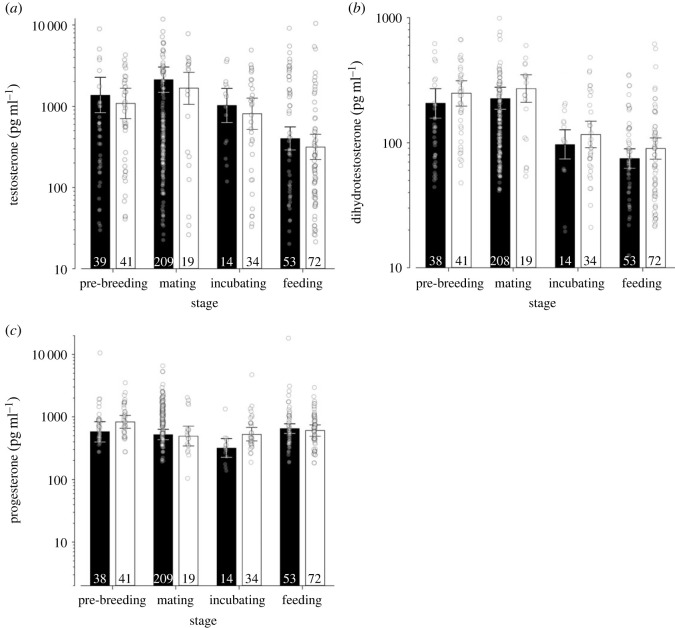
(*a*) Testosterone, (*b*) DHT and (*c*) progesterone concentrations of male black coucals (black bars) and male white-browed coucals (open bars; see figure 1 for details of the graph properties). Please note the logarithmic scaling of the *y*-axes.

In males, testosterone concentrations did not differ between the two species. Testosterone concentrations were highest during the mating phase, intermediate during pre-breeding and incubation, and lowest when feeding nestlings (figure 2*a*; table 2*a*; electronic supplementary material, table S2). Similar to testosterone, DHT levels of males did not differ between species; they were highest during pre-breeding and mating compared to incubation and when feeding nestlings (figure 2*b*; table 2*b*; electronic supplementary material, table S2). Overall, progesterone concentrations of males did not differ between the two species, but in black coucals progesterone concentrations were lower during incubation than during all other stages (all *P*(β)’s > 0.99) and lower than those of white-browed coucals in the same stage (*P*(β) = 0.99; figure 2*c*; table 2*c*; electronic supplementary material, table S2).

**Table 2.** Comparison of male black and white-browed coucals (WBC). Estimates of effect sizes and the 2.5% and 97.5% quantiles (limits of the 95% Bayesian credible interval) of the posterior distribution of log hormone concentrations (for details on *P*(β) see table 1).

parameter	posterior mean	2.5 % quantile	97.5 % quantile	*P*(β)
(*a*) testosterone (*R*² = 0.218, adj *R*² = 0.204; *F*_4,222_ = 15.440).				
intercept (pre-breeding)	7.219	6.725	7.732	1
species (WBC)	−0.237	−0.613	0.139	0.890
stage mating	0.437	−0.134	1.031	0.934
stage incubating	−0.292	−0.292	0.299	0.827
*stage feeding*	*−1.225*	*−1.774*	*−0.704*	*0.999*
(*b*) DHT (*R*² = 0.318, adj *R*² = 0.305; *F*_4,224_ = 26.07).				
intercept (pre-breeding)	5.332	5.057	5.600	1
species (WBC)	0.183	−0.025	0.400	0.959
stage mating	0.085	−0.229	0.411	0.704
*stage incubating*	*−0.762*	*−1.078*	*−0.436*	*0.999*
*stage feeding*	*−1.019*	*−1.297*	*−0.733*	*0.999*
(*c*) progesterone (*R*² = 0.103, adj *R*² = 0.074; *F*_7,220_ = 3.597).				
intercept (pre-breeding)	6.363	5.985	6.729	1
species (WBC)	0.363	−0.078	0.803	0.947
stage mating	−0.103	−0.523	0.310	0.311
*stage incubating*	*−0.606*	*−1.120*	*−0.093*	*0.989*
stage feeding	0.117	−0.294	0.524	0.712
stage × species				
** **WBC mating	−0.419	−1.010	0.176	0.916
** **WBC incubating	0.148	−0.467	0.774	0.686
** **WBC feeding	−0.432	−0.953	0.073	0.952

### (b) Gonadotropin-releasing hormone-induced steroid hormone concentrations and testosterone increase

**Figure 3.**
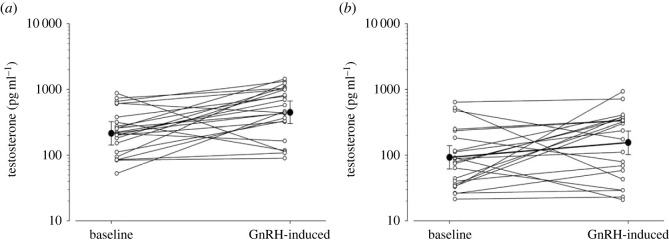
Testosterone response to intramuscular injections of 7.5 µg GnRH in (*a*) female black and (*b*) female white-browed coucals. Large filled circles and error bars refer to posterior means and their respective 95% credible intervals, small open circles refer to data points of individual females.

Intramuscular injection of 7.5 µg GnRH led to an increase in testosterone in female black and white-browed coucals. Baseline and GnRH-induced testosterone concentrations were higher in female black coucals than white-browed coucals, but the slope of the increase did not differ between species (figure 3; table 3*a*). Both baseline testosterone and GnRH-induced testosterone response decreased with Julian day (table 3*a*). GnRH injections also increased progesterone and corticosterone concentrations of female coucals, but did not affect DHT concentrations (see electronic supplementary material).

**Table 3.** Baseline and GnRH-induced testosterone comparison of black and whited-browed coucals (for a legend see table 1).

parameter	posterior mean	2.5 % quantile	97.5 % quantile	*P*(β)
(*a*) female testosterone (marg. *r*² = 0.349, cond. *r*² = 0.375; random eff.: female ID 0.033 ± 0.182, residual var. 0.816 ± 0.903).				
intercept	5.378	4.978	5.777	1
*species (WBC)*	*−0.851*	*−1.420*	*−0.267*	*0.998*
*GnRH*	*0.720*	*0.166*	*1.286*	*0.994*
species × GnRH	−0.201	−0.998	0.609	0.309
*Julian day*	*−0.287*	*−0.500*	*−0.077*	*0.996*
(*b*) male testosterone (marg. *R*² = 0.231, cond. *R*² = 0.548; random eff.: male ID 0.788 ± 0.888, resid var. 1.126 ± 1.061).				
intercept	5.663	5.144	6.204	1
species (WBC)	−0.377	−1.092	0.344	0.847
*stage: mating*	*1.670*	*1.062*	*2.247*	*1*
*stage: incubating*	*1.303*	*0.483*	*2.101*	*0.999*
GnRH	0.373	−0.182	0.920	0.908
species × GnRH	−0.062	−0.806	0.670	0.957
(*c*) male DHT (marg. *R*² = 0.250, cond. *R*² = 0.354; random eff.: male ID 0.170 ± 0.412, resid var. 1.051 ± 1.025).				
intercept	4.121	3.692	4.556	1
species (WBC)	0.358	−0.189	0.935	0.891
*stage: mating*	*1.108*	*0.634*	*1.565*	*1*
stage: incubating	−0.098	−0.695	0.493	0.376
*GnRH*	*1.142*	*0.594*	*1.688*	*1*
*species × GnRH*	*−0.924*	*−1.629*	*−0.208*	*0.996*

**Figure 4.**
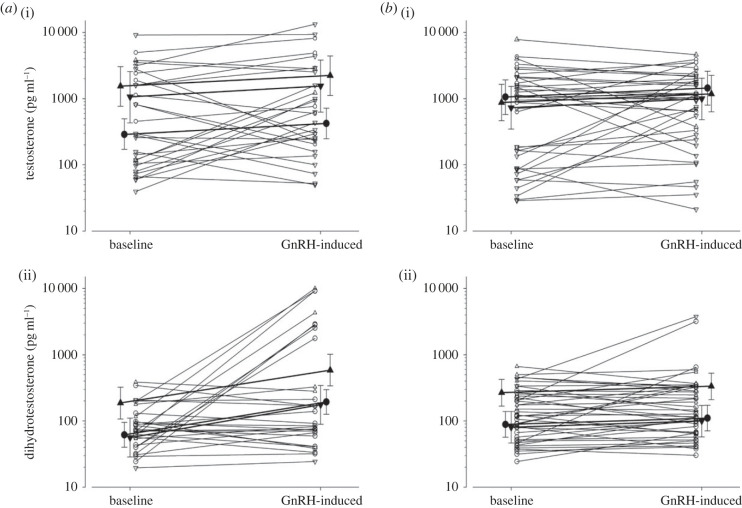
Testosterone (i) and DHT response (ii) to intramuscular injections of GnRH in (*a*) male black and (*b*) male white-browed coucals. Upward- and downward-facing triangles refer to mating and incubating birds, respectively, and circles to offspring-feeding birds; the large symbols and their error bars refer to posterior means and their 95% credible intervals.

In males, injections of 7.5 µg GnRH did not substantially increase circulating levels of testosterone, but mating and incubating males had higher levels of baseline testosterone and GnRH-induced concentrations of testosterone than males that were feeding offspring (figure 4; table 3*b*). In contrast with testosterone, GnRH injections led to an increase in DHT in black coucals but not in white-browed coucals (figure 4; table 3*c*). Progesterone and corticosterone concentrations increased in males of both species (see electronic supplementary material).

## 4. Discussion

Baseline and GnRH-induced testosterone concentrations of classically polyandrous female black coucals were higher than those of monogamous female white-browed coucals. Thereby, this study provides the first evidence that testosterone concentrations of females in a sex-role-reversed species may be higher than those of a closely related species with Darwinian sex roles. Progesterone concentrations of female black coucals were lowest during the pre-breeding phase, when they established their territories after arriving at the breeding grounds. In contrast with females, baseline and GnRH-induced testosterone concentrations did not differ between males of both species. Progesterone was lowest in incubating male black coucals, a pattern that reflects that of females in birds with Darwinian sex roles (e.g. [93]).

The differences in testosterone and progesterone concentrations between female black coucals and female white-browed coucals support the hypothesis that these hormones modulate sex-role-reversed traits (albeit the sample size is relatively low for pre-breeding and mating white-browed coucals). Female black coucals' higher testosterone levels correspond to a high testosterone sensitivity of their *nucleus taeniae*, a brain structure involved in the regulation of reproductive and aggressive behaviour [50,94], suggesting that testosterone modulates sex-role-reversed behaviours of females. This role of testosterone is, however, far subtler than a naïve expectation of a complete sex reversal in hormone secretion pattern would suggest. Instead of completely reversing circulating concentrations to high 'male-like' levels, testosterone seems to be rather slightly elevated in female black coucals with concentrations that are approximately twice as high as those of female white-browed coucals. Such a nuanced change in baseline and GnRH-inducible testosterone concentrations could—in combination with the higher sensitivity for the hormone in relevant brain areas [50]—safeguard females against potentially harmful effects of high, 'male-like' concentrations of this hormone. A number of studies showed that experimental manipulation of testosterone in females can delay breeding, interfere with mate choice, affect parental care or modulate the immune system [32,95–99]. However, it is currently unclear if high circulating levels of testosterone in the physiological range would elicit such effects. Alternatively, the higher testosterone concentrations are not cause, but the consequence of differences in behaviour (see [100] for an example in male barn swallows). Agonistic interactions with other females do not elevate testosterone in female black coucals [77], but perhaps frequent mating interactions with males elevate testosterone, as has been shown for male birds (reviewed by [101]).

Low levels of progesterone in pre-breeding female black coucals are consistent with an experimental study, in which progesterone concentrations of female black coucals dropped during simulated territorial intrusions, and in which progesterone implants led to a reduction in territorial aggression compared to control-implanted females [77], a situation similar to female rats [102]. Females continue to lay eggs during the whole mating period which can last for several months. Slightly elevated levels of progesterone during this period are consistent with findings from other bird species during egg-laying (e.g. [93,103]). It is therefore rather surprising that, in white-browed coucals, progesterone levels tended to be higher during the pre-breeding period than during the mating period. Low levels of progesterone in the incubation phase of male black coucals are consistent with low-progesterone concentrations of incubating females of other species [93,103,104]. However, the hormonal regulation of incubation behaviour requires a suite of interacting hormones [104] and further studies including measurements and experimental manipulations of sex steroids and prolactin in males would be required to elucidate the hormonal regulation of incubation behaviour in male coucals. Moreover, in white-browed coucals, incubation is shared by males and females, but also in this species males typically have a larger share in incubation than females [72].

Females of both species responded with an elevation of testosterone and progesterone to stimulation with GnRH, suggesting that they can respond to hypothalamic stimulation with an increase in steroid levels. A similar responsiveness of females to GnRH stimulation has been found in dark-eyed juncos (*Junco hyemalis* [105–107]) tree swallows (*Tachycineta bicolor* [108]) and non-breeding northern cardinals (*Cardinalis cardinalis* [109]). The results also show huge individual differences both in baseline and GnRH-induced steroid hormone levels, which could reflect differences in the regulation and fitness-relevance of hormone-mediated traits, such as mating behaviour and fertility. Since simulated territorial intrusions did not elevate testosterone in female black coucals [77], it is unlikely that aggressive territorial interactions stimulate testosterone release. Hence, interactions with mating partners, non-social environmental or intrinsic factors may be responsible for the variation in hormone levels among individuals. Individual variation in hormone concentrations has been recognized to be potentially important (e.g. [110–112]), but practically it remains challenging to address the relevance of such individual differences—in particular in field studies, where repeated sampling of hormone levels is usually difficult. The GnRH stimulation data of the current study are in contrast with previous work, where female black coucals did not elevate testosterone in response to GnRH injections [44]. However, this earlier study had used a lower dose of GnRH (0.5 µg versus 7.5 µg), which was injected intravenously into only eight females. Hence, low dosage and a small sample size may explain why the earlier study did not find an effect.

In contrast with male white-browed coucals, male black coucals have only one functional testis [81]. Nevertheless, baseline and GnRH-induced testosterone concentrations were similar in males of both species. Obviously, the lack of one testis does not seem to reduce the production of testosterone and therefore is unlikely to represent a physiological mechanism for the reversal of sex roles, as initially suggested by Ligon [113]. Male black coucals regularly sire young in other males’ nests, but they do so mainly within the ‘harem’ of the female they are partnered with [78]. Hence, from a male black coucal's perspective much of this behaviour does not really qualify as ‘extra-pair’ behaviour and therefore has a different ‘flavour’ than the typical extra-pair behaviour of other species, where males seek to mate and sire offspring with females outside their own pair-bond. Thus, high rates of extra-pair paternity may be related to testosterone concentrations in males of species in which males seek extra-pair copulations with females other than their partner [59], but not in classical polyandrous black coucals, where males mainly sire young in the nests of other males which are partnered with the same female.

Testosterone concentrations in males of both species were lowest during the parenting period, in line with experimental evidence that high levels of testosterone suppress paternal care in many birds (summarized by [57,58]). The lack of an overall increase in testosterone after stimulation with GnRH in males of both species is in contrast with an earlier study in which six male black coucals (all measured during the nestling feeding period) consistently elevated testosterone after GnRH stimulation with a smaller intravenous dose of GnRH [44]. However, another androgen, DHT, was responsive to GnRH injections also in the current study: DHT concentrations of male black coucals, but not male white-browed coucals, increased after GnRH injections, suggesting that the GnRH injection had some effect on androgens. Both testosterone and DHT bind to the androgen receptor, so that an increase in circulating concentrations after a GnRH injection can act also via DHT and not only testosterone.

To the best of my knowledge, this is the first description of differences in sex steroid concentrations of two closely related species with monogamous and classical polyandrous mating systems. This represents a two-species comparison, an approach that has been discouraged because it statistically confounds two independent variables: species membership and the factor in question [65,114], in this case different mating systems including a reversal in sex roles. For this reason, Garland *et al*. [65,114] consider phylogenetic comparative studies more appropriate also for physiological studies. However, one major disadvantage of such larger scale comparisons of endocrine mechanisms across species (and laboratories) is the fact that hormone measures are rarely standardized across laboratories, thereby introducing substantial variability or 'noise' in the measurement [64]. The large variability of hormone measures across laboratories [63] renders it difficult to interpret comparative hormone data, especially when differences or effect sizes are small: existing differences may be hidden by method-introduced 'noise'. So far, comparative studies did not find differences in testosterone concentrations between socially monogamous and classical polyandrous bird species [33,52–54]. Ketterson *et al*. [31] even found that females of socially monogamous species have higher levels of testosterone than females of species with other mating systems, but they did not account for phylogeny. The current study demonstrates consistent differences in testosterone concentrations between female black and female white-browed coucals, but the differences occur at low overall levels, i.e. with mean testosterone concentrations of female black coucals of roughly 250 pg ml^−1^, being twice as high as those of female white-browed coucals with 120 pg ml^−1^. These concentrations were measured with the same radioimmunoassay and within the same laboratory with an inter-assay variation of 12.8% for testosterone. Assuming a 5- to 10-fold error range of measurements across laboratories [63], such a onefold difference between classical polyandrous and monogamous species would have probably been masked by the overall variability in testosterone concentrations of females in a comparative study across species and laboratories.

Pending further evidence, the current study suggests that female black coucals have slightly elevated testosterone concentrations compared to female white-browed coucals, and that this elevation may therefore play a role in regulating sex-role-reversed behaviours. Comparative data from other coucals species (measured in the same laboratory) would be needed to confirm this finding. But what about other bird or vertebrate species with similar mating systems? Can we assume elevated testosterone to represent a universal mechanism for higher female competition for access to gametes, mating partners or parental care providers? So far, comparative studies comparing mating systems across birds, did not find any evidence for this, but—as stated above—this could be due to a low signal-to-noise ratio because of the larger variability in hormone measurements across laboratories. However, it could also be that different taxa evolved different mechanisms [64,115]. Within birds, coucals, buttonquails, some sandpiper species (*Scolopacidae*) and jacanas have evolved classical polyandry independently. Most likely, androgens such as testosterone play a role in regulating relevant morphological or behavioural traits, but they may do so in different ways. The hormonal signalling cascade is complex and according to our current understanding signalling mechanisms and traits are more likely to be able to evolve independently rather than in tandem. This means that selection may act on traits such as hormone levels, hormone receptor expression, cofactor expression, and clearance of hormones independently from one another to affect a regulatory outcome [25,116]. Female black coucals and button quail, for instance, apparently increased their brain sensitivity for testosterone [50,51], and in black coucals, this increase in sensitivity went along with overall higher female testosterone concentrations compared to white-browed coucals. The testosterone production in response to GnRH stimulation, however, was similar in females of both coucals species, suggesting that the hormonal cascade of the HPG axis has not been modified. It is unknown, though, if other classical polyandrous species use the same mechanism. Perhaps they only increased the sensitivity of target areas in the brain without altering circulating testosterone levels. Comparative testosterone data of closely related species with different mating systems or sex roles measured in the same laboratory might help to answer this question.

Recently, Lipshutz & Rosvall [34] reviewed the literature on classical polyandrous bird species and found that the difference in testosterone concentrations between females and males disappears during parenting. Males of classical polyandrous species have higher levels of testosterone during mating, but in contrast with biparental species or species with female-only care, sex differences in testosterone disappear during parental care [34]. The current study demonstrates that this finding does not hold for black coucals: even males that incubate or feed offspring had levels of testosterone that are substantially higher than those of females (figures 1 and 2; electronic supplementary material, tables S1 and S2). Possibly, this has to do with the fact that male black coucals do not drop out of the mating pool once they incubate eggs or feed nestlings, but instead continue siring offspring in other males’ nests [80]. Also, male black coucals frequently receive replacement clutches and can raise more than one clutch per season, so that their reproductive axis remains active throughout the breeding season [72]. The fertile period may differ across species with a classical polyandrous mating system and needs to be considered in future comparisons.

In summary, the current study suggests that androgens may aid amazons, and there is some indication that progesterone may promote good pas. This is exciting because it hints at conserved functions of testosterone and progesterone in systems with reversed sex roles compared to systems with Darwinian sex roles. To consolidate this finding, we need further paired comparisons of closely related species that differ in sex roles and, given suitable model species, experimental studies that either manipulate hormone concentrations or alter sex roles to see which effects hormones have on behaviour and vice versa.

## Data Availability

Data for this article are available via Dryad (doi:10.5061/dryad.f1vhhmh1d) [117]. Supplementary material is available online [118].
